# Microbial ecology of the atmosphere

**DOI:** 10.1093/femsre/fuac009

**Published:** 2022-02-08

**Authors:** Tina Šantl-Temkiv, Pierre Amato, Emilio O Casamayor, Patrick K H Lee, Stephen B Pointing

**Affiliations:** Department of Biology, Aarhus University, DK-8000 Aarhus, Denmark; Stellar Astrophysics Centre, Department of Physics and Astronomy, Aarhus University, DK-8000 Aarhus, Denmark; Institut de Chimie de Clermont-Ferrand, SIGMA Clermont, CNRS, Université Clermont Auvergne, 63178, Clermont-Ferrand, France; Centre for Advanced Studies of Blanes, Spanish Council for Research (CSIC), 17300, Blanes, Spain; School of Energy and Environment, City University of Hong Kong, Hong Kong, China; State Key Laboratory of Marine Pollution, City University of Hong Kong, Hong Kong, China; Yale-NUS College, National University of Singapore, Singapore 138527; Department of Biological Sciences, National University of Singapore, Singapore 117558

**Keywords:** aeromicrobiology, bioaerosols, microbial biogeography, microbial dispersal, microbial ice nucleation, One Health

## Abstract

The atmosphere connects habitats across multiple spatial scales via airborne dispersal of microbial cells, propagules and biomolecules. Atmospheric microorganisms have been implicated in a variety of biochemical and biophysical transformations. Here, we review ecological aspects of airborne microorganisms with respect to their dispersal, activity and contribution to climatic processes. Latest studies utilizing metagenomic approaches demonstrate that airborne microbial communities exhibit pronounced biogeography, driven by a combination of biotic and abiotic factors. We quantify distributions and fluxes of microbial cells between surface habitats and the atmosphere and place special emphasis on long-range pathogen dispersal. Recent advances have established that these processes may be relevant for macroecological outcomes in terrestrial and marine habitats. We evaluate the potential biological transformation of atmospheric volatile organic compounds and other substrates by airborne microorganisms and discuss clouds as hotspots of microbial metabolic activity in the atmosphere. Furthermore, we emphasize the role of microorganisms as ice nucleating particles and their relevance for the water cycle via formation of clouds and precipitation. Finally, potential impacts of anthropogenic forcing on the natural atmospheric microbiota via emission of particulate matter, greenhouse gases and microorganisms are discussed.

## Teaser

This review identifies ecological drivers of microbial distribution in the atmosphere, evidence for their involvement in biochemical and biophysical transformations, and risks from anthropogenic forcing.

## Introduction

An implicit assumption in microbial ecology is that extensive atmospheric transport between habitats occurs due to the aerosolization and long-range transport of microorganisms (Womack *et al*. 2010, Zhou and Ning 2017). At any time, the atmosphere supports vast and highly variable numbers of microbial cells, pollen, propagules, cell fragments, viruses and biomolecules (Després *et al*. 2012), which account for primary biological aerosols (bioaerosols) estimated to represent 25% of all particles larger than 200 nm (Jaenicke 2005). Most cells in the atmosphere are metabolically inactive during transport between source and sink destinations and experience a high turnover (Burrows *et al*. 2009). The fate of viable bioaerosols is important to maintenance of diversity and resilience in terrestrial and marine ecosystems globally (Womack *et al*. 2010, Hanson *et al*. 2012, Barberan *et al*. 2014). Bioaerosols also have important health-related impacts via the dispersal of animal (Lai *et al*. 2009) and plant (Brown and Hovmøller 2002) pathogens, gene flow of antibiotic resistance genes (Pal *et al*. 2016) and microbially derived allergens (Woo *et al*. 2013). In addition, the recognition that some microorganisms mediate biochemical (Deguillaume *et al*. 2008) and biophysical transformations (Morris *et al*. 2008) during brief residence periods in the atmosphere offers the prospect of an active microbial contribution to atmospheric processes.

**Figure 1.**
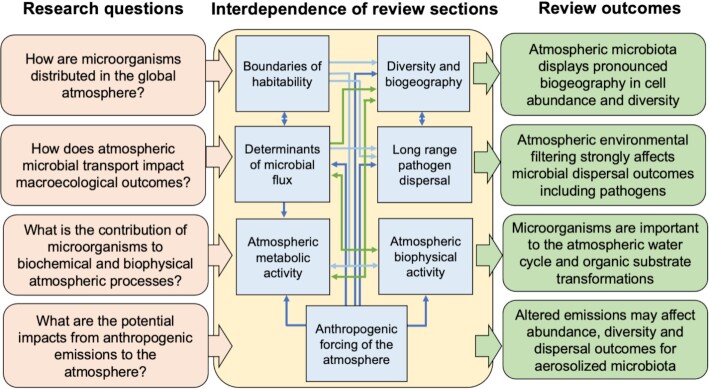
Structure of this review. Schematic of the research questions and outcomes of this review, with the interdependence of topics within the review indicated by arrows. Arrow colours are for clarity of viewing and do not reflect different processes.

In this review, the unique complexity of the atmospheric environment and its microbiota is identified and the extent and limitations to current understanding of its ecological relevance are considered (Fig. 1). The scope encompasses microorganisms that occur in outdoor atmospheric air for extended periods or travel across regional or inter-continental distances. The person-to-person transmission of human pathogens facilitated by exhaled respiratory droplets and bioaerosols in air, e.g. SARS-CoV-2 (Kutter *et al*. 2018, Leung 2021), and human-associated microorganisms suspended in dust within the indoor built environment (Hospodsky *et al*. 2012) are excluded. This is because they involve brief residence times for microorganisms in air and defining the nature, extent and importance of aerosol transmission for these scenarios is still under active discussion (Leung 2021). Specific focus is given to how insights from molecular ecological studies have identified atmospheric microbiology as key to understanding the interdependence of ecosystems via flux of diverse taxa between atmosphere and surface habitats, and critical examination of the genetic and physiological evidence for microbial activity in the atmosphere. Furthermore, because the atmosphere is facing profound change due to anthropogenic emissions, consideration is given to how this may impact its microbiology in future.

## Boundaries for habitability in the atmosphere

**Figure 2.**
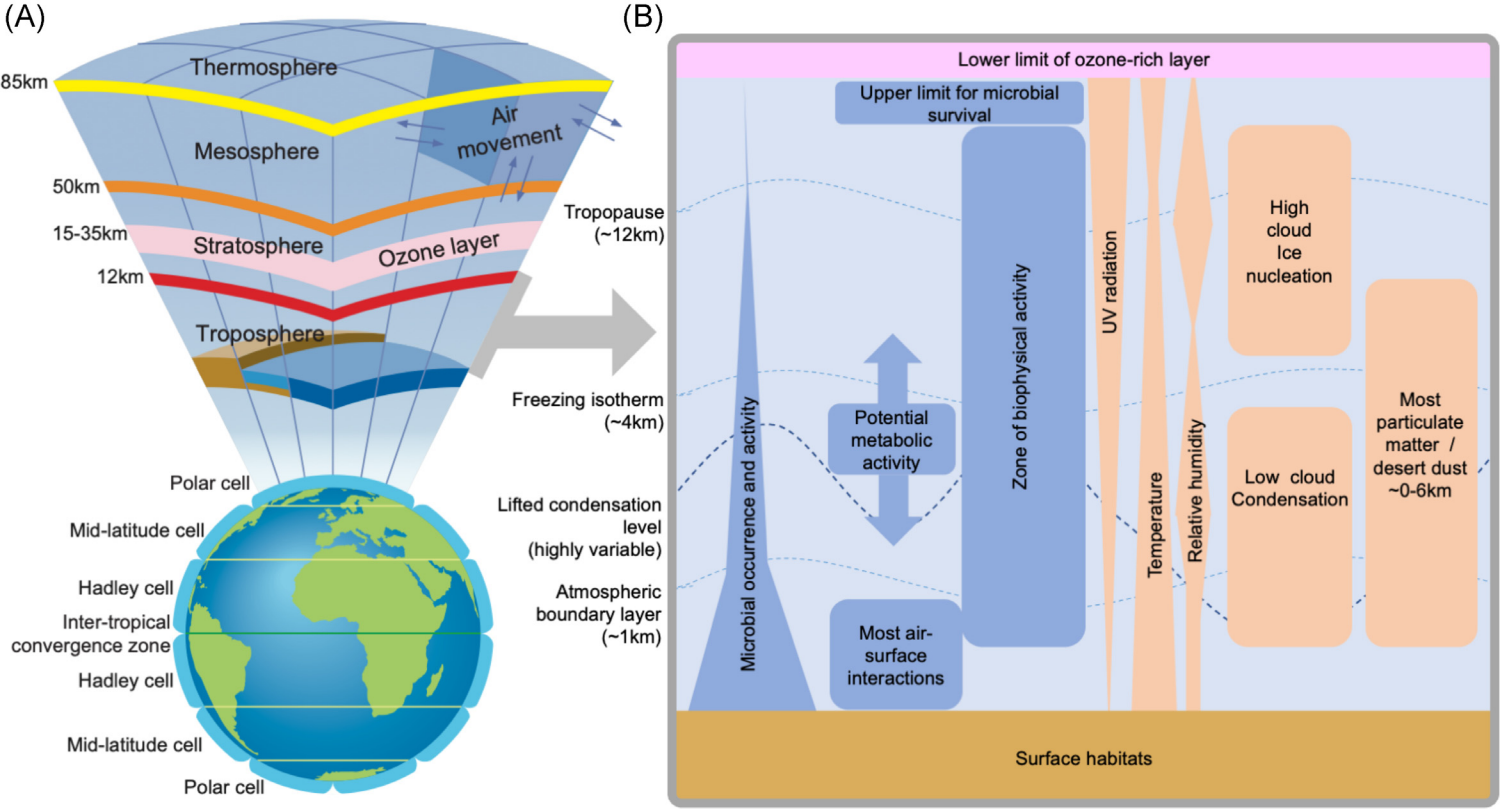
Defining the atmospheric ecological niche. **(A)** The atmosphere comprises a series of vertically delineated concentric layers that are primarily defined by thermal properties. The troposphere contains 75% of the molecular and gaseous mass of the atmosphere and almost all of the water vapour, clouds and aerosolized particles including microorganisms. **(B)** Four boundaries that occur at varying altitude within the troposphere are important in terms of atmospheric microbial ecology: The atmospheric boundary layer delineates the region of air closest to the surface where the bulk of surface–atmosphere interactions occur, and this includes exchange of microorganisms with terrestrial and marine ecosystems. The lifted condensation level (LCL) delineates the layer above which cloud formation occurs. It is variable with temperature and humidity and delineates the thermal limit for condensation of saturated water vapour on particulate surfaces. The freezing isotherm delineates the layer above which freezing occurs and is also the limit for active non-psychrophilic microbial metabolism. We note that the relationship between the atmospheric boundary layer, the LCL and the freezing isotherm, which are highly dynamic, is significantly more complex than presented in the figure, e.g. both the LCL and the freezing isotherm can sit at ground level well within the boundary layer. The boundary between the LCL and freezing isotherm represents the theoretical range for mesophilic microbial metabolic activity, with potential for psychrophilic and xerophilic activity to extend beyond this zone. The tropopause marks the upper limit of the troposphere and mixing with upper layers is limited by the thermal inversion in the stratosphere. The bulk of atmospheric ozone is located in the lower stratosphere and this likely delineates the altitudinal limit of survival for many atmospheric microorganisms. Blue shapes denote biotic attributes and orange boxes denote physical attributes.

The atmosphere comprises a series of vertically delineated concentric layers whose altitude and depth are primarily defined by thermal properties (Fig. 2A). The troposphere is the lowermost atmospheric layer that contains 75% of its molecular and gaseous mass as well as most water vapour and atmospheric particles (Barry and Chorley 2010), as well as the majority of microbial cells (Fig. 2B). This is where water cycles through clouds, precipitation and surface environments. It includes the atmospheric boundary layer (a.k.a. planetary boundary layer) that is in direct contact with the surface, and the overlaying free troposphere that is of great relevance for regional and global dispersal due to long-range movement of air masses. Vertical mixing above the troposphere is limited due to thermal inversion. Above the troposphere, the bulk of atmospheric ozone is located in the lower stratosphere and this is likely to represent the altitudinal limit to survival for the majority of microbial cells. This is due to lethal UV exposure at higher altitudes as a function of reduced attenuation by ozone and extended residence time for suspended biological particles at higher altitudes (Bryan *et al*. 2019).

The troposphere presents an extremely physico-chemically challenging but nonetheless potentially habitable environment for microorganisms. The presence of sufficient liquid water in cells is a fundamental prerequisite for any microbial activity and is subject to atmospheric relative humidity that is determined by physical conditions and evapotranspiration. In the troposphere, relative humidity is generally bimodal, with higher values in the lower and upper troposphere and a drier mid-troposphere (Fig. 2B) (Gettelman *et al*. 2006). The LCL is the altitude at which vapour pressure of an air parcel becomes saturated and above the LCL, at slight supersaturations, the water vapour will condense on aerosol particles and form clouds. The LCL varies widely depending on the temperature and water vapour content, which fluctuate with latitude, location and time, determining the cloud patterns (Fig. 2B). Microbial exploitation of low moisture in xeric environments (Pointing and Belnap 2012, Lebre *et al*. 2017) suggests that sub-saturated relative humidity levels throughout the troposphere are not inhibitory to adapted microbial taxa although the lower limit of relative humidity that supports active metabolism has not been determined. In contrast, only regions of high relative humidity and clouds are likely to support most microorganisms not specifically adapted to low water activity. Cloud droplets provide aqueous microenvironments (Hill *et al*. 2007, Šantl-Temkiv *et al*. 2013a, Joly *et al*. 2015), also the thin layer of liquid water that occurs at the surface of bioaerosols under subsaturated conditions may be sufficient to sustain microbial activity (Stevenson *et al*. 2015a).

Photo-oxidative and thermal stress are among other major challenges to microbial survival and proliferation in the troposphere (Fig. 2B). A strong altitude-dependent radiation gradient occurs in the troposphere due to the attenuation by ozone, oxygen, water vapour and particulates, and overall UV-A and UV-B radiation increases ∼10–20% with every km in altitude (Barry and Chorley 2010). Whilst UV radiation exposure *per se* is a strong environmental filter to microbial survival and influences diurnal bacterial occurrence in air (Tong and Lighthart 2006), it is unlikely to be a limiting factor to radiation-tolerant taxa. Air temperature decreases with altitude in the troposphere at the rate of ∼6.5°C per km from ambient near-surface temperatures to −50°C or lower at the tropopause (NOAA, NASA and US Air Force 1976) in the absence of inversions. The freezing isotherm occurs at variable altitudes between ground level and ∼5 km according to season, latitude, terrain and atmospheric conditions (Fig. 2B). This delineates the mechanism of precipitation formation and the altitudinal limit for non-psychrophilic microbial metabolism. Microbial metabolism has been demonstrated at temperatures below those encountered even in the uppermost region of the troposphere (Amato and Christner 2009), and so whilst the freezing isotherm undoubtedly represents a physiological boundary, temperature *per se* is unlikely to inhibit potential microbial metabolism or survival in the troposphere.

A peculiarity of the atmosphere contrasting with other environments such as soils and oceans is its highly dynamic nature with high cell turnover rates (Burrows *et al*. 2009). Temperature, humidity and liquid water content can change within minutes, exposing cells to repeated thermal and osmotic shock, and freeze–thaw cycles during residence in air. Additionally, as air density decreases with altitude so does the spatial proximity of cells and their potential substrates. The limited availability of surfaces in the atmosphere is also a challenge to microbial community development as it limits conventional biofilm formation and the possibility for microbial interactions, e.g. via quorum sensing (Monier and Lindow 2003). Overall, the atmosphere has been described as possessing qualities of a chaotic system in terms of its physicochemical complexity (Hochman *et al*. 2019), and this underlines its heterogeneity as a microbial environment.

## Diversity and biogeography of atmospheric microorganisms

**Figure 3.**
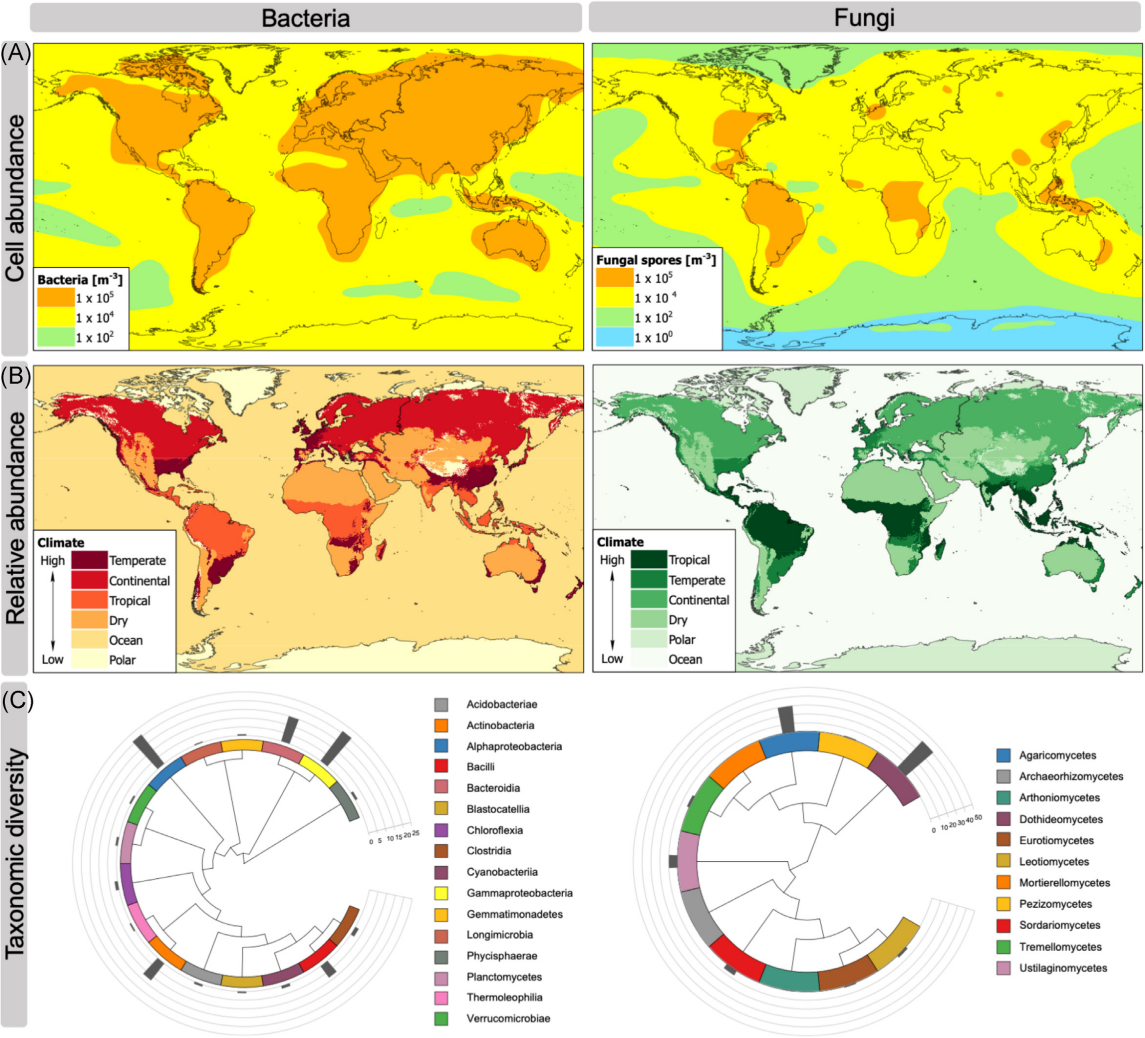
Patterns of microbial diversity and abundance in the atmosphere. **(A)** Simulated concentration of bacterial cells and fungal spores in the near surface atmospheric boundary layer, adapted from Hoose, Kristjánsson and Burrows (2010). Models assumed a uniform bacterial cell diameter of 1 µm and fungal spore diameter of 5 µm (Burrows *et al*. 2009), and emission rates were estimated from observed and modelled data (Burrows *et al*. 2009, Heald and Spracklen 2009). **(B)** Predicted global relative abundance of bacteria and fungi in the near-surface atmospheric boundary layer from observed quantitative PCR of rRNA genes in air above locations within different climatically defined biomes (Archer *et al*. 2022). The use of quantitative PCR data is cautiously interpreted here as relative abundance and is not used to infer cell numbers. These data are also domain specific and cannot be directly compared between bacteria and fungi. **(C)**Taxonomic composition by class of bacteria and fungi in the near-surface atmospheric boundary layer inferred from rigorously decontaminated environmental rRNA gene sequence data from globally distributed locations, adapted from Archer *et al*. (2022). Coloured boxes indicate classes encountered at a relative abundance of ≥0.1%. Grey bars indicate percentage relative abundance for each class.

The physical volume of the troposphere dwarfs that of the equivalent microbially inhabited layers of the ocean (i.e. the photic zone) or land (i.e. topsoil) although microbial abundance is several orders of magnitude lower (Whitman *et al*. 1998, Bar-On *et al*. 2018). On a global scale, modelled atmospheric cell concentrations fall within the range of 10^0^–10^5^ per m^3^ for bacteria and fungi (Burrows *et al*. 2009, Hoose *et al*. 2010, Spracklen and Heald 2014), and observed geographic patterns in relative abundance of cells are broadly congruent with modelled estimates of their biogeography (Mayol *et al*. 2017, Tignat-Perrier *et al*. 2019, Archer *et al*. 2022) (Fig. 3A and B). Generally, the major delineation in global microbial abundance occurs between air masses above terrestrial and marine ecosystems, with concentration of bacterial and fungal cells highest above terrestrial surfaces and decreasing markedly with distance from land (Mayol *et al*. 2017). Direct cell counting (Mayol *et al*. 2017), estimates from sequencing of environmental DNA (Bryan *et al*. 2019, Gusareva *et al*. 2019, Tignat-Perrier *et al*. 2019, Archer *et al*. 2022) and historical cultivation approaches (Després *et al*. 2012) collectively indicate that the most abundant cellular microorganisms within the atmospheric boundary layer are bacteria and fungi. Other microbial groups such as archaea and protists appear to comprise a relatively minor component in air (Cáliz *et al*. 2018, Gusareva *et al*. 2019, Archer *et al*. 2022). Estimates from fresh snowfall and from cloud water suggest 4 × 10^2^–4 × 10^3^ bacterial cells per m^3^ and 4 × 10^0^–4 × 10^2^ eukaryotic cells per m^3^ assuming condensed water content of 0.4 g per m^3^ (Christner *et al*. 2008b, Tesson *et al*. 2016, Amato *et al*. 2017, Hu *et al*. 2018, Šantl-Temkiv *et al*. 2019). Cells associated with aerosolized desert dust may reach 10^7^ per m^3^ (Maki *et al*. 2016). Aggregations of cells that may be free-floating or attached to the surface of particles may create localized elevated concentrations of biomass. In this regard microorganisms within clouds and particulate dust plumes are likely to be significant. The global total mass of clouds has been estimated at 194 000 Tg (Pruppacher and Jaenicke 1995), and the fraction of the atmosphere under cloud cover has been estimated at 67% globally (King *et al*. 2013). Up to 2 billion tonnes of desert dust is transported in the atmosphere annually (Shepherd *et al*. 2016), and a comparable mass of soil dust particulates are also emitted to the atmosphere by arable lands every year (Borrelli *et al*. 2017). Attempts to extrapolate the various estimates in order to estimate global atmospheric cell abundance have a high level of uncertainty and have not been able to adequately encompass variability between boundary layer, troposphere, stratosphere, cloud and dust-free regions, clouds and dust. Nonetheless, a global scale meta-analysis of bacterial abundance across biomes indicated ∼5 × 10^19^ cells may occur globally in the atmospheric boundary layer, compared with ∼1.2 × 10^29^ in oceans and 2.6 × 10^29^ in soils globally (Whitman *et al*. 1998). This estimate was several orders of magnitude lower than those for ocean, soil and subsurface biomes, and reflects the extremely low density of cells in the atmosphere. The number of viruses in the atmosphere is uncertain but may be significant (Reche *et al*. 2018). Cell-free microbial metabolites and cell fragments also constitute a significant portion of suspended particulate organic matter in air, above both terrestrial and ocean surfaces (Wilson *et al*. 2015, Tobo *et al*. 2019).

Microbiological diversity estimations for the atmosphere have been strongly influenced by methodological biases (Šantl-Temkiv *et al*. 2020). Direct comparisons of cultivation versus molecular ecological approaches have indicated very few atmospheric microorganisms are cultivable (Temkiv *et al*. 2012), and this is supported by order of magnitude higher diversity estimates from molecular ecology surveys (Cáliz *et al*. 2018, Archer *et al*. 2019, 2020, 2022, Tignat-Perrier *et al*. 2019, Uetake *et al*. 2020). The recent emergence of portable high-volume air sampling devices has greatly helped in recovery of sufficient biomass for diversity estimation with sample sizes of several hundred cubic metres of air now possible during short timeframes (Šantl-Temkiv *et al*. 2020). A persistent issue for diversity estimation has been in distinguishing genuine microbial signatures from the unavoidable contaminants that occur from equipment and reagents when interrogating ultra-low biomass samples. Assessment of contamination during sequence-based diversity estimation indicates that at the very low sample DNA template concentrations encountered in air, reagent-derived contaminants may form a significant component of sequenced diversity (Salter *et al*. 2014). For this reason a rigorous approach to mitigation is required during experimental and bioinformatic workflows (Eisenhofer *et al*. 2019). Therefore, it is also necessary to strongly nuance findings where decontamination effort and outcome were not fully reported.

Nonetheless, a growing body of recent research indicates a consensus in the understanding of microorganisms in bulk air (i.e. including free cells, aggregates and particulate-associated cells). Taxa adapted to survival under high UV irradiance, moisture limitation and other challenges are over-represented in atmospheric communities, which is indicative of strong environmental filtering. This is supported by evidence that atmospheric microbial communities exhibit distinct phylogenetic structuring and elevated abundance of stress-tolerant taxa within the boundary layer and at higher altitudes (Tignat-Perrier *et al*. 2019, Archer *et al*. 2022). Thus theoretical frameworks in ecology that have made implicit assumptions about ubiquitous microbial distribution in air are being validated and revised (Dumbrell *et al*. 2010, Lowe and McPeek 2014, Zhou and Ning 2017).

Across broad geographic, altitudinal and temporal scales in the troposphere taxonomic diversity broadly reflects recruitment from underlying surface source habitats, and commonly encountered bacteria affiliate with the classes Alphaproteobacteria, Gammaproteobacteria, Bacteroidia, Actinobacteria and Bacilli (Fig. 3C). Spore-forming bacteria are particularly prevalent and also taxa adapted to environmental stresses encountered in the atmosphere, e.g. desiccation, oxidative stress (Aalismail *et al*. 2019, Archer *et al*. 2022). The commonly encountered fungal classes in air largely comprise Dothideomycetes and Agaricomycetes (Cáliz *et al*. 2018, Gusareva *et al*. 2019, Tignat-Perrier *et al*. 2019, Els *et al*. 2019b, Archer *et al*. 2022) (Fig. 3C). These are highly diverse fungal groups mostly encountered in the air as spores rather than hyphae. The estimation of atmospheric viral diversity is an emerging topic with few studies to date: Across a range of urban, coastal and forest land use types, the most abundant DNA viruses recovered belonged to the ssDNA *Gemini-*,*Circo-*,*Nano-* and *Microviridae* (Whon *et al*. 2012). The study recovered lower abundance of dsDNA viruses but acknowledged that methodological limitations may have affected recovery for this group (Whon *et al*. 2012). In another study Geminiviruses associated with the phyllosphere and plant diseases were the most abundant group (García-Arenal and Zerbini 2019). A laboratory simulation of virus aerosolization from marine sea spray concluded that the *Polydnoviridae* and *Alloherpesviridae* viruses were enriched in aerosols (Michaud *et al*. 2018). The RNA viruses were not targeted in these ecological studies and so uncertainties remain about their distribution in free air.

Biogeographic regions for atmospheric microorganisms have been proposed based upon global air circulation patterns of latitudinally defined air cells (Fig. 2A) (Womack *et al*. 2010). This is supported by recent evidence for regionalization (Uetake *et al*. 2020) and biogeographic patterns in atmospheric bacterial and fungal diversity on a global scale (Archer *et al*. 2022). Furthermore, these studies point towards a highly complex biogeography that also reflects surface habitats and climatic factors. A high microbial species richness within a given air mass in the atmospheric boundary layer reflects recruitment from a variety of terrestrial and marine sources, and source tracking has indicated combined influence of local and distantly recruited taxa to observed diversity (Cáliz *et al*. 2018, Archer *et al*. 2022). Diversity estimates for near-ground air within the atmospheric boundary layer at various locales have indicated bacterial and fungal communities that were correlated with local abiotic variables such as temperature, precipitation and humidity or land use/surface cover, reflecting the role of local cell emissions (Bowers *et al*. 2011, Fröhlich-Nowoisky *et al*. 2012, Tignat-Perrier *et al*. 2019, Archer *et al*. 2022, Spring *et al*. 2021). Thus, diversity of fungi is relatively higher above regions with a well-developed phyllosphere (Lymperopoulou *et al*. 2016, Archer *et al*. 2022, Zhou *et al*. 2021), and lowest above oceans (Mayol *et al*. 2017, Archer *et al*. 2020, Uetake *et al*. 2020) and low productivity terrestrial systems such as drylands and polar/alpine regions where underlying surface communities are less diverse and microbially dominated by bacteria (Archer *et al*. 2019, 2022). Several studies have also related diversity to factors associated with dispersal such as history of air masses and UV radiation, suggesting combined influence of predominant remote sources and conditions during transit (Bowers *et al*. 2012, Cáliz *et al*. 2018, Archer *et al*. 2019, Uetake *et al*. 2019, Els *et al*. 2019b, 2020, Tignat-Perrier *et al*. 2020).

Temporal patterns in local atmospheric microbiota at a specific location reflect seasonal changes in underlying surface cover and the trajectory of incoming air masses. Inter-seasonal variation in the atmospheric boundary layer was found to be absent (Gusareva *et al*. 2019), weak (Tignat-Perrier *et al*. 2020), pronounced for some taxa (Bowers *et al*. 2012) or stochastic (Els *et al*. 2019b), depending on the location. Still, a large number of studies have identified differences in bacterial and/or fungal communities in summer versus winter at temperate and sub-tropical terrestrial locations (Bowers *et al*. 2012, 2013, Woo *et al*. 2013, Barberán *et al*. 2014, Cáliz *et al*. 2018, Uetake *et al*. 2019, Els *et al*. 2019b). Two of the scarce continuous long-term studies demonstrated a clear and well-delineated seasonal pattern in bacterial and fungal communities at near-ground locations (Woo *et al*. 2013) and above the atmospheric boundary layer (Cáliz *et al*. 2018). At polar latitudes elevated cell abundance in air corresponded to summer with snow-free terrestrial surfaces (Šantl-Temkiv *et al*. 2019). Patterns may also occur over shorter timeframes, with a pronounced diel variation of specific bacterial and fungal groups observed for the equatorial tropics (Gusareva *et al*. 2019). Together these studies strengthen the view that the underlying phyllosphere is a major determinant of atmospheric microbial diversity as they are related to patterns of plant ontogeny and growing season. As the elevation above ground increases, the source area of influence widens (Schmid 2002, Hsieh and Katul 2009), and the small-scale spatial and temporal heterogeneities observed near the ground blur (Archer *et al*. 2019, Els *et al*. 2019a, 2022). Above the atmospheric boundary layer long-range air movements progressively decouple atmospheric diversity from its emission sources (Burrows *et al*. 2009) (Smith *et al*. 2018).

Whilst most research has focused on the troposphere and specifically the atmospheric boundary layer, understanding the biogeographic limit of microbial occurrence and survival at higher altitudes is also of interest. It has been postulated that limited microbial survival and transport may occur in the stratosphere (DasSarma and DasSarma 2018), but the role of the stratosphere in microbial dispersal may be limited due to the combined effects of extreme environmental stress and extended residence times (Bryan *et al*. 2019). Several studies have employed balloon and aircraft platforms to recover stratospheric aerosol samples and they have described a limited diversity and viability of bacterial and fungal taxa (Wainwright *et al*. 2003, Smith *et al*. 2010, 2011, 2018, Bryan *et al*. 2019), with taxa not markedly differentiated from those occurring in the troposphere below (Smith *et al*. 2018). Even though estimates of abundance vary greatly a recent study suggested that bacterial abundance in the troposphere and lower stratosphere may be comparable (Bryan *et al*. 2019). However, there is currently insufficient evidence to draw robust conclusions about the occurrence, transport or viability of microorganisms in the stratosphere. The discipline of astrobiology has also considered the potential role of the stratosphere and upper atmosphere in the potential interplanetary dispersal of microorganisms as free cells or via mineral vectors (Yang *et al*. 2009) but this will require major investigative effort to be further considered.

## What are the determinants of microbial flux between the atmosphere and surface habitats?

The atmosphere has long been identified as a major conduit for microbial transport between surface habitats, e.g. Womack *et al*. (2010), and this has a profound impact on the function and resilience of terrestrial and marine surface communities due to its contribution to gene flow, community assembly and dispersal of pathogens (Zhou and Ning 2017). Recent developments in ecological theory have begun to challenge the long-held view that microbial transport is a neutral process (Lowe and McPeek 2014), and recent evidence from large-scale biogeographic (Mayol *et al*. 2017, Archer *et al*. 2022) and temporal (Cáliz *et al*. 2018, Tignat-Perrier *et al*. 2020) studies of atmospheric microbiota increasingly support this view. Deterministic influence due to environmental filtering and microbial stress tolerance both play a significant role in shaping atmospheric microbiota (Archer *et al*. 2019, 2022), and hence the composition of assemblages that are exchanged between atmosphere and surface habitats.

**Figure 4.**
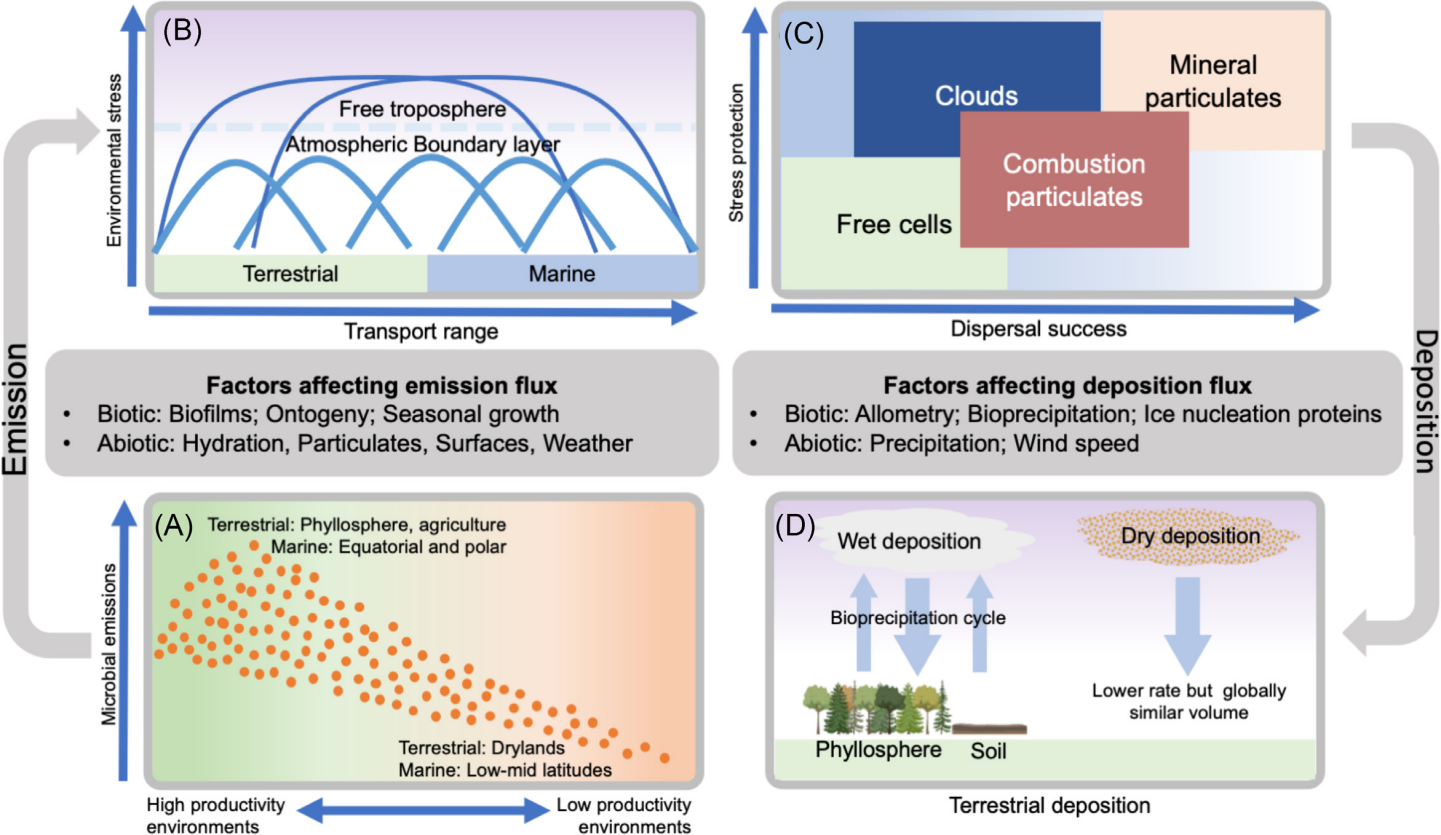
Factors affecting atmospheric microbial transport and macroecological outcomes. **(A)** Emission and recruitment of microorganisms to the atmosphere are strongly dependent on the underlying surface habitats. This includes major delineations between terrestrial and marine locations, and a strong influence of productivity in underlying habitats. Biotic traits may also be important to emission variations between taxa. **(B)** Above terrestrial and marine surfaces at local scales transport occurs largely within the atmospheric boundary layer over relatively short distances. At larger spatial scales microbial transport involves transit above the boundary layer and this results in longer residence times and greater environmental filtering.**(C)** The association of cells with clouds or particulates directly impacts survival and transport. Combustion particulates may be less efficient vectors than mineral particles from desert dust and soil because they co-aerosolize with lower numbers of microorganisms, and they are associated with toxic combustion products. **(D)** Deposition of cells from the atmosphere occurs via wet deposition as rainfall, hail and snow, and via dry deposition that relies on sedimentation of cells due to gravity and occurs at lower velocities than wet deposition. Wet deposition over continents is typically induced by ice nucleation, which leads to preferential deposition of ice nucleation active cells and contributes to bioprecipitation and other feedbacks.

The major determinants of microbial fluxes in the atmosphere between source and sink habitats are emission, survival during transport and deposition (Fig. 4). Emission from the surface, known as aerosolization, is dependent upon the substrate and a major factor is the nature of the interface with air, such as the composition of plant cover (Zhou *et al*. 2021), cohesiveness of soil (Joung *et al*. 2017, Archer *et al*. 2022) or chemistry of the water-surface microlayer (Michaud *et al*. 2018) (Fig. 4A). Emissions from terrestrial and marine environments are higher when productivity of the underlying surface environment is greater (Burrows *et al*. 2009) (Fig. 4A). The processes affecting microbial emissions are highly variable and subject to stochastic influence from events such as dust storms (González-Toril *et al*. 2020) and wildfires (Moore *et al*. 2021). Biotic microbial traits that affect aerosolization are also important, including cell hydrophobicity and membrane composition (Burger and Bennett 1985, Michaud *et al*. 2018), extracellular polymeric substance (EPS) production (Morris and Monier 2003), spore production and release (Lagomarsino Oneto *et al*. 2020), and allometry (Norros *et al*. 2014). For viruses the possession of a hydrophobic envelope has been linked with a tendency to aerosolize and to desiccation tolerance (Michaud *et al*. 2018). Modelling emissions for particulates 1–3 µm diameter as a proxy for single and particle-associated bacterial cells revealed that the total annual emissions were in the range of 7.6 × 10^23^–3.5 × 10^24^ cells largely originating from terrestrial environments, i.e. crops, grasslands and shrubs (Burrows *et al*. 2009, 2013). This equated to an estimated annual emission of 470–1100 Gg bacterial biomass (Burrows *et al*. 2013). Fungal emissions occur predominantly as free spores and were estimated at 9.5 × 10^23^ total spores from terrestrial surfaces per year, which amounts to ∼50 Tg of fungal biomass annually (Elbert *et al*. 2007). The emission rate of viral particles from surface habitats is currently uncertain.

The major marine aerosolization process is initiated by breaking waves that produce small air bubbles that scavenge microorganisms and organic compounds from the sea column and transport them to the sea surface (Carlucci and Williams 1965, Aller *et al*. 2005), where they burst to eject small droplets containing salts, biogenic material and microorganisms into the atmosphere (Blanchard and Syzdek 1970, Blanchard 1989, Afeti and Resch 1990). Even though oceans are thought to represent weak sources of airborne microorganisms on a global per surface area basis, bioaerosol emissions include bacteria and viruses (Aller *et al*. 2005, Wilson *et al*. 2015, Mescioglu *et al*. 2019, Uetake *et al*. 2020) and selective transfer and enrichment of organic compounds in marine aerosols has been shown experimentally (Rastelli *et al*. 2017). In addition, large spatial and temporal variability in marine emissions and effects on atmospheric processes may occur due to marine biological processes and meteorological factors (Wang *et al*. 2015, Wilson *et al*. 2015, DeMott *et al*. 2016).

**Figure 5.**
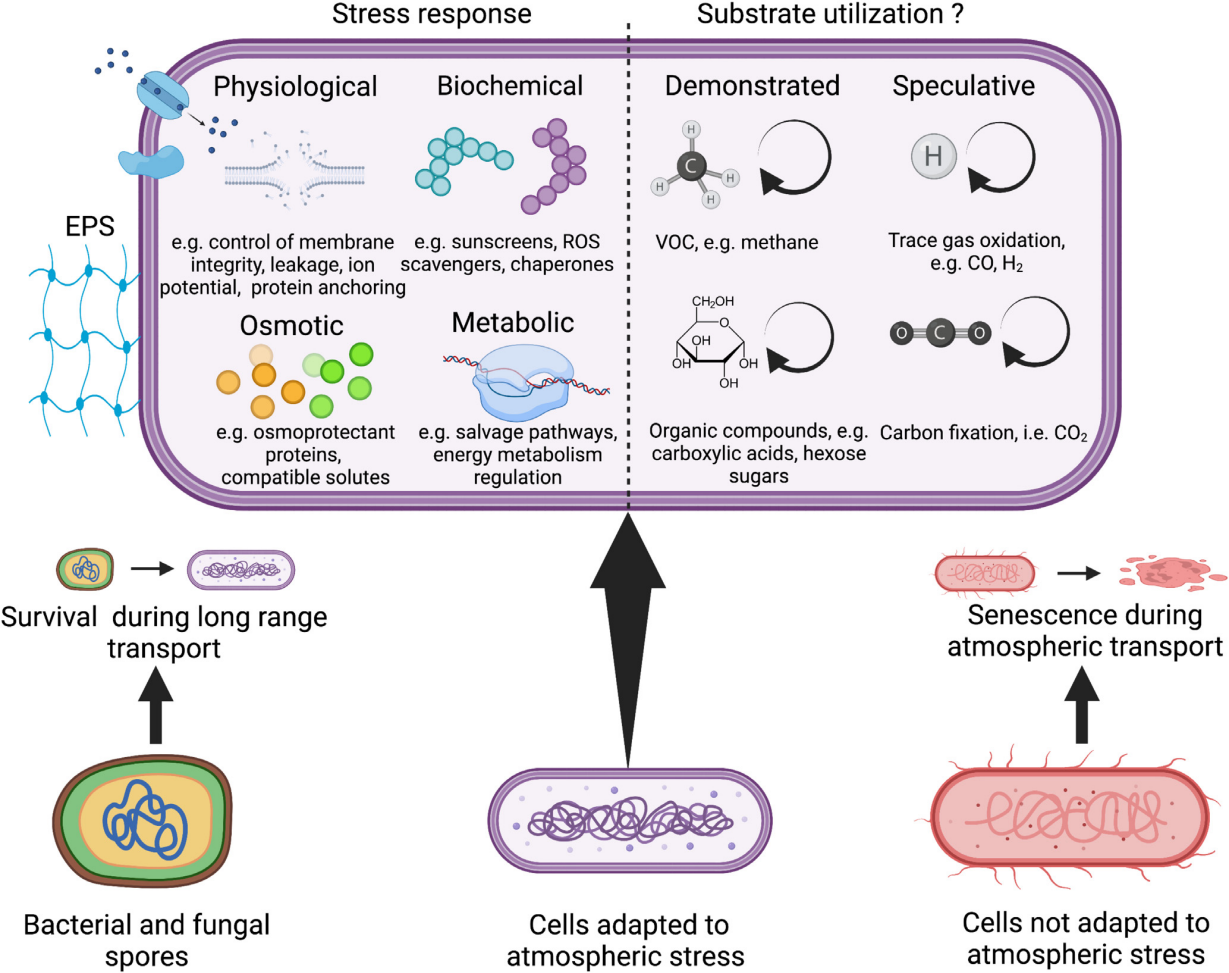
Stress response and potential substrate utilization by atmospheric microorganisms. Cells that possess adaptation to atmospheric environmental stressors are potentially capable of metabolic activity during atmospheric transport. A range of intracellular protective and repair strategies to reactive oxygen species (ROS) and other stressors may be complemented by extracellular protection due to EPSs that also confer protection against xeric, thermal and ultraviolet radiation (UVR) stress. Atmospheric bacteria oxidize volatile organic compounds (VOC) such as methane and a range of small organic compound carboxylic acids, pentose and hexose sugars, amino acids and phenolic compounds. Oxidation of other atmospheric trace gases and carbon fixation may also be potential pathways for activity in the atmosphere although supporting data are currently lacking.

Microbial transport fluxes are affected by the altitude that the cells reach, which is a deterministic factor related to stress exposure (Fig. 4B). Survival during transit in air is influenced by biotic traits involved in stress tolerance, and particularly with regard to mitigating the effects of low water activity (Stevenson *et al*. 2015b), desiccation (Potts 1994), photo-oxidative UV (Daly 2009, Sharma *et al*. 2017), general oxidative stress (Ziegelhoffer and Donohue 2009, Ezraty *et al*. 2017) and low temperatures/freezing (Amato 2013) (Fig. 5). Dormancy and sporulation are strategies that provide protection against major stressors and functional metagenomics of air has indicated genes associated with these traits are over-represented in atmospheric communities compared with underlying surface habitats (Aalismail *et al*. 2019, Archer *et al*. 2022). Many spores as well as dormant and active cells possess pigment compounds such as melanins, mycosporines and scytonemins that protect against oxidative and desiccation stress largely due to scavenging of reactive species (Gao and Garcia-Pichel 2011). A number of biodiversity surveys in air have revealed taxa known to produce protective pigments are over-represented in air compared with underlying terrestrial and marine habitats (Fröhlich-Nowoisky *et al*. 2012, Tignat-Perrier *et al*. 2019, Archer *et al*. 2022), which implies a survival advantage. Additional strategies to counter stress may include the secretion of EPSs to mitigating both low water activity via hygroscopic carbohydrate polymers as well as provide extracellular protection from oxidative stress (Matulová *et al*. 2014). Aerosolization of cells as aggregates, biofilms or attached to particulates may also confer survival advantages during dispersal (Monier and Lindow 2003, Morris and Monier 2003) (Fig. 4), but may also reduce residence time in air due to increased size and density (Burrows *et al*. 2009).

Metagenomic profiling of atmospheric communities has begun to reveal prevalence of genes encoding cold shock, oxidative stress and UV repair enzymes (Aalismail *et al*. 2019, Archer *et al*. 2022), and metatranscriptomics of cloud water has revealed active metabolic responses to counter oxidative, osmotic and thermal stresses (Amato *et al*. 2019). Physiological responses against simulated atmospheric stress have also been observed for taxa isolated from the atmosphere. Strains of *Pseudomonas syringae* isolated from rain and cloud water withstood aerosolization, freezing, osmotic shock, short-term exposure to UVA and UVB radiation (Alsved *et al*. 2018, de Araujo *et al*. 2019, Ling *et al*. 2021), and oxidative stress imposed by hydrogen peroxide (H_2_O_2_) (Wirgot *et al*. 2019). Metabolomic studies on *P. syringae* also revealed a suite of responses to cold shock under simulated atmospheric conditions (Jousse *et al*. 2017), including production of cryoprotectants, antioxidants, alkaloids and metabolites involved in energy metabolism. The persistence of viruses in aerosols and traits that facilitate survival is not well constrained at this time (Leung 2021). However, a laboratory simulation has shown that a number of ssDNA, dsDNA and RNA viruses can remain viable in an aerosolized form across a range of temperature, humidity and UV exposures (Verreault *et al*. 2015). The infectivity of airborne viruses towards microorganisms suspended in the atmosphere is unknown and further investigation is required to determine if such interactions may impact survival during transport.

A very large fraction (97%) of depositing bacterial cells have been found to be particle associated (Reche *et al*. 2018), and with a large mean diameter (∼9 μm) (Woo and Yamamoto 2020), thus supporting the idea that the mixing state of cells in air, i.e. free cells, aggregates or cells vectored on particulate matter, has a significant effect on cell residence time (Fig. 4C). The deposition of microorganisms occurs through a combination of wet and dry deposition (Després *et al*. 2012). The wet deposition is affected by a combination of in-cloud partitioning during cloud droplet/ice particle formation and below-cloud aerosol scavenging processes, which can both contribute to segregation of taxa between the wet and dry phases (Bauer *et al*. 2003, Moore *et al*. 2020, Woo and Yamamoto 2020). In particular, cloud condensation ability was modelled to shorten the average bacterial residence time in air (and hence dispersal distance) from ∼8 to 3 days (Burrows *et al*. 2009) and ice nucleating microbial cells were shown to be efficiently precipitated (Amato *et al*. 2015, Stopelli *et al*. 2015). Wet deposition rates of 10^7^–5 × 10^9^ bacteria cells m^−2^ d^–1^ have been reported that exceed dry deposition (Reche *et al*. 2018, Woo and Yamamoto 2020). Dry deposition velocity is largely determined by allometric considerations, such as biological particle size, shape and density, as well as meteorology (Fig. 4D). Globally the volume of wet and dry deposition may be similar due to the frequency of precipitation events versus dry periods (Reche *et al*. 2018, Woo and Yamamoto 2020). Patterns in deposition of fungal spores and pollen are thought to follow a similar pattern although taxon-specific variation is likely due to spore characteristics (Aylor 2017, Woo and Yamamoto 2020). Deposition of viruses from the free troposphere has been estimated at 0.26 × 10^9^ to >7 × 10^9^ viral particles/day and was positively correlated with fine organic aerosols (<0.7 μm) but displayed no significant variation between wet and dry deposition (Reche *et al*. 2018).

A pertinent question for atmospheric microbial communities is the extent to which the complex variation in emission and deposition fluxes, together with environmental filtering during transit, influence the assembly of terrestrial (including freshwater) and marine surface communities. The flux of biomass containing diverse taxa from air to land or oceans is likely smaller by magnitudes than that of standing communities but given the ability of microorganisms to rapidly exploit favourable ecological niches, atmospheric flux should be viewed as a critical control point in aspects of connectivity and ecological resilience across biomes (Dumbrell *et al*. 2010, Lowe and McPeek 2014, Zhou and Ning 2017). This occurs by facilitating microbial dispersal between spatially separated natural systems (Kellogg and Griffin 2006), contributing to maintenance of diversity via recruitment (Jones and Lennon 2010), promoting dispersal of dormant propagules that may act as microbial seed banks (Lennon and Jones 2011) and dispersal of phages that exert critical control on microbial population turnover (Sandra *et al*. 2004).

## What is the evidence for long distance atmospheric pathogen dispersal?

The person to person transmission of pathogens over short distances in air is well documented and covered in excellent recent reviews (Kutter *et al*. 2018, Leung 2021). Human respiratory pathogens and particularly viruses are transmitted by a variety of means that include respiratory droplets (≥0.1 mm diameter) that are rapidly deposited from air, and respiratory aerosols (≤0.1 mm diameter) that may remain suspended for longer periods and thus disperse over longer distances up to several metres (Leung 2021). Current knowledge on the extent and relative importance of this short-range person to person aerosol transmission is currently not well constrained, e.g. Kutter *et al*. (2018) and Leung (2021). Airborne genetic signatures of human bacterial and viral pathogens have also been detected in aerosols of confined built environments with point source emissions such as livestock facilities, e.g. Nehme *et al*. (2008) and Zhao *et al*. (2014), and indoor wastewater treatment plants, e.g. Yang *et al*. (2019). Such bioaerosols appear to be highly localized and evidence for significant transport in free atmospheric air beyond point sources is lacking. These scenarios are significant for public health but there is currently a lack of compelling evidence that they pose risk associated with long-range atmospheric transport.

Long-range atmospheric dispersal of pathogens (i.e. over hundreds to thousands of kilometres) typically requires protection from atmospheric environmental stress as evidenced by the taxonomy and physiological state of recovered taxa, e.g. Mayol *et al*. (2017), Cáliz *et al*. (2018) and Šantl-Temkiv *et al*. (2018). Despite this, some evidence exists for regional dispersal of pathogens up to hundreds of kilometres without any obvious adaptation to environmental stress during atmospheric transport, e.g. foot and mouth diseases virus (Sorensen *et al*. 2000, Björnham *et al*. 2020). More typically long-range pathogen dispersal involves preadaptation due to the formation of spores or other resting structures (Fröhlich-Nowoisky *et al*. 2009), possession of stress tolerance traits such as oxidative stress avoidance and repair pathways (Archer *et al*. 2022), and/or association with particulate vectors (Maki *et al*. 2019). Vectoring on biological surfaces also provides a protected means of long-distance dispersal: Wind-borne transport of *Anopheles* mosquitoes carrying the *Plasmodium* parasite was recorded over several hundred kilometres in the Sahel region of Africa (Huestis *et al*. 2019), and pollen has been demonstrated as a vector for bacteria and bacterial allergens (Oteros *et al*. 2019). Correlations have been made between regional trans-continental scale atmospheric dust vectoring and pathogen dispersal (Griffin 2007, Tobias *et al*. 2019). Consistent inter-annual dynamics for specific taxa may exist that make them foreseeable over time with dominance of plant pathogens over those of animals and humans (Triadó-Margarit *et al*. 2022).

Fungal spores are particularly well suited to long-range dispersal and are problematic as a threat to agriculture and global food security (Brown and Hovmøller 2002, Savary *et al*. 2019). Many plant pathogens have historically displayed distinct regionalization but globalization of agriculture has exacerbated range shifts (Bebber *et al*. 2014). Airborne fungal pathogens have been detected above land with agricultural land use (Nicolaisen *et al*. 2017, Archer *et al*. 2022), and some fungal pathogens are able to disperse across inter-continental distances and reestablish infections even when host plants are seasonally absent (Brown and Hovmøller 2002). A striking example is the reemergence of wheat stem rust as a threat to global food supply. The disease caused by the basidiomycete fungus *Puccinia graminis* f. sp. *tritici* is dispersed readily in air by basidiospores. It was virtually eradicated in the twentieth century but a highly pathogenic new strain, Ug99 and its variants, emerged in the last decade and have threatened to devastate global wheat cultivation (Singh *et al*. 2011). Combination of disease surveillance and dispersal modelling has resulted in strong evidence for atmospheric transmission as a major determinant of wheat rust epidemiological zones (Meyer *et al*. 2017).

Evidence for long-range atmospheric dispersal of human pathogens also exists. Seasonal outbreaks of meningococcal meningitis in sub-Saharan Africa have been correlated with bacterial vectoring on dust (Jusot *et al*. 2017), and this also appears to occur for fungal diseases such as Valley Fever caused by *Coccidioides* spores transmitted on desert dust across the southwestern United States and northern Mexico (Kollath *et al*. 2019), and across the Atlantic Ocean to Europe (Triadó-Margarit *et al*. 2022). In addition to human pathogens, atmospheric transport of microbially derived allergens (Woo *et al*. 2013) and antibiotic resistance genes (Pal *et al*. 2016, Li *et al*. 2018, Caliz *et al*. 2022) may pose health risks to human, agricultural and natural populations (Bai *et al*. 2022). Atmospheric microbiology is therefore relevant to the One Health conceptual framework that seeks to highlight how the wellbeing of humans, animals, plants and the environment are inter-connected (CDC 2020). Emission, transport and deposition of microorganisms via the atmosphere at various spatial scales are central to some relationships identified by this framework. It is envisaged as important to improved understanding of the transboundary aspect of how pathogens, antibiotic resistance and invasive taxa traverse regions and exert negative outcomes.

## What is the evidence for metabolically active atmospheric microorganisms?

The majority of cells in the atmosphere are unlikely to be metabolically active but are simply undergoing transport between source and sink destinations with a high turnover (Burrows *et al*. 2009). Given that most fungi in air occur as dormant spores it is likely that the active fraction of atmospheric microbiota is comprised almost exclusively of bacteria that possess adaptation to atmospheric environmental stress (Archer *et al*. 2022) (Fig. 5). Putative generation times for bacteria in cloud water have been estimated at 3.6–19.5 days (Sattler *et al*. 2001), which fits within their modelled airborne residence times (Burrows *et al*. 2009). However, whether metabolic transformations *in situ* under the highly fluctuating atmospheric conditions are sufficient to sustain only quasi-dormancy, or if cell homeostasis and reproduction are energetically feasible within the short timeframe of favourable conditions during atmospheric transport remains unexplored.

Evidence from environmental samples has demonstrated that atmospheric microorganisms possess the potential to transform substrates encountered in the atmosphere (Fig. 5). Molecular ecological surveys of cloud water (Amato *et al*. 2019), free air (Gusareva *et al*. 2019, Archer *et al*. 2022) and airborne desert dust plumes (Aalismail *et al*. 2019) indicate diverse autotrophic and heterotrophic metabolic pathways in atmospheric assemblages that broadly reflect source environments. Photosynthesis may be limited in atmospheric microorganisms due to light inhibition (Barber and Andersson 1992), but clouds and desert dust provide shading against damaging levels of direct UV radiation, and diverse viable and stress-tolerant microalgae and cyanobacteria have been reported from clouds (Tesson and Šantl-Temkiv 2018). A range of pathways indicating atmospheric trace gas metabolism including hydrogen, methane and carbon monoxide were widespread among atmospheric metagenomes (Archer *et al*. 2022) and methane oxidation at atmospheric concentrations has been demonstrated by *Methylocystis* and *Methylosinus* enriched from the atmosphere (Šantl-Temkiv *et al*. 2013b), although it is unclear if this is energetically feasible under atmospheric conditions. Most research, however, has focused on the potential for heterotrophic metabolism of simple organic compounds in clouds as these are envisaged as ‘hotspots’ for atmospheric microbial activity.

Microbial assemblages in cloud water have revealed general markers of metabolic activity under simulated atmospheric conditions, including ATP, high ribosomal content, transcribed RNA, and enzymatic and respiratory activities (Klein *et al*. 2016, Šantl-Temkiv *et al*. 2018, Amato *et al*. 2019). Under conditions of liquid water and substrate availability, favourable temperature and reduced stress exposure, atmospheric microorganisms appear capable of active heterotrophic metabolism as evidenced by chemical speciation in recovered cloud water (Vaitilingom *et al*. 2013, Bianco *et al*. 2019). A comparative metagenomic and metatranscriptomic study of cloud water assemblages highlighted that active metabolism in clouds is closely coupled with satisfying the energetic demands of active stress response metabolism and particularly with regard to mitigating oxidative stress (Amato *et al*. 2019). A complex metabolism was envisaged that also included synthesis of cryoprotectant and EPSs implicated in a protective role. The study identified C1 and C2 compounds as important substrates and ammonium as the major nitrogen source (Amato *et al*. 2019). Another combined metatranscriptomic and metabolic study of cloud water provided strong evidence for phenol metabolism by bacteria, and this was envisaged as a possible atmospheric substrate derived from anthropogenic emissions (Lallement *et al*. 2018).

Several studies have demonstrated the ability of atmospheric isolates to metabolize compounds encountered in clouds: A screening of 60 atmospheric isolates including the bacterial genera *Bacillus*, *Pseudomonas*, *Micrococcus* and *Sphingomonas* as well as fungal yeasts from cloud water revealed that under laboratory conditions all were capable of degrading diverse carboxylic acids and volatile organic compounds (Amato *et al*. 2007). Atmospheric bacterial isolates of *Bacillus* sp., *Frigoribacterium* sp., *Pseudomonas* sp. and the methanotrophic genera *Methylocystis* sp. and *Methylosinus* sp. have been demonstrated to oxidize common atmospheric volatile organic compounds including formaldehyde and methanol (Husárová *et al*. 2011, Šantl-Temkiv *et al*. 2013b). *Rhodococcus enclensis* isolated from cloud water was shown to metabolize catechol and phenol under simulated atmospheric conditions at rates comparable to that of chemical transformations in the atmosphere (Jaber *et al*. 2020). Mono- and disaccharides may also serve as substrates, as demonstrated for a *Bacillus* strain isolated from cloud water (Matulová *et al*. 2014).

Based on bulk cloud water measurements, which were unable to account for the multiphase structure and highly dynamic nature of clouds, it has been proposed that heterotrophic microbial activity could be the main driver for the oxidation of common atmospheric organic compounds in air during night and could compete with photochemistry during the day (Vaïtilingom *et al*. 2010). Modelling has indicated that given the small fraction (∼10^–4^) of spatially separated microdroplets that contain microbial cells, the quantitative contribution of microbial activity to the transformation of atmospheric compounds is likely to be low (Fankhauser *et al*. 2019), although this needs to be verified with experimental approaches simulating *in situ* atmospheric conditions. More detailed modelling, accounting for the *in situ* cloud structure, has suggested that even though the biodegradation of highly water-soluble organic gasses, such as dicarboxylic acids, may have been overestimated, the biodegradation of organic gases with intermediate solubility, such as acetate and formate, is more efficient and may represent a significant sink for these compounds (Khaled *et al*. 2021).

## What is the microbial role in atmospheric biophysical processes?

**Figure 6.**
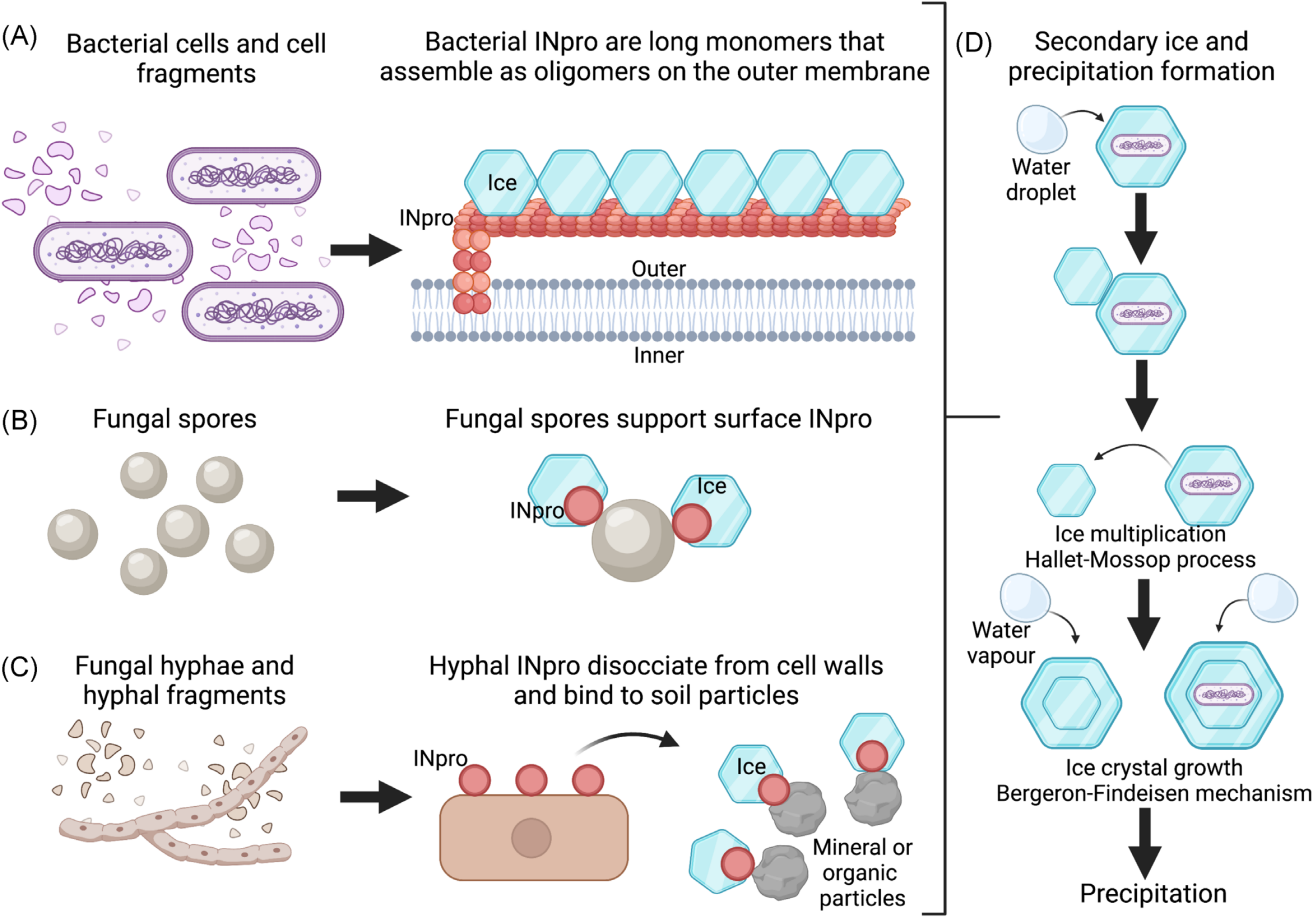
Biophysical role of atmospheric microorganisms in ice nucleation. An important biophysical interaction of atmospheric microorganisms with cloud formation is ice nucleation by specific microbial proteins that contributes to precipitation and the hydrological cycle, as well as impacting microbial deposition from the atmosphere to surface habitats. **(A)**Bacterial ice nucleating proteins (INpro) are long repetitive proteins that are anchored on the surface of the outer membrane and nucleate ice through a yet to be fully resolved mechanism. They may be cell associated or retain activity in cell fragments. Monomers or small oligomers nucleate ice at temperatures from −7 to −10°C and higher order oligomers of INpro form at well-defined sizes that nucleate ice between −2 and −5°C. Fungal spores and hyphae are associated with INpro that exhibit different molecular structure and properties than those in bacteria, but they are yet to be fully described. **(B)** Fungal spores are the dominant state for fungi in the atmosphere and support surface-associated INpro and ice nucleating activity. **(C)** Fungal hyphae occur very rarely in the atmosphere and so the major contribution arises from fungal INpro that are easily dissociated from cell walls and adhere to soil particulates that subsequently become aerosolized. **(D)** Low concentrations of atmospheric INpro nucleate cloud ice and may induce subsequent secondary ice formation via the Hallet–Mossop process. This process involves freezing of supercooled cloud droplets that encounter primary ice particles (riming) and subsequent ejection of frozen droplets that form independent ice particles (splintering). Primary and secondary ice particles form precipitation through the Bergeron–Findeisen mechanism where ice growth occurs at the expense of surrounding water droplets. Images in panels (A–D) are not to scale: in panels (A–C) hexagonal shapes represent ice embryos forming on INpro and in panel (D) hexagonal shapes represent ice particles in clouds.

Microbial involvement in biophysical transformations occurs independently from the metabolic activity that is thought to be rare in the atmosphere. Biological ice nucleation contributes to cloud formation and precipitation, and more generally to climate and the hydrological cycle. It also impacts microbial flux between atmospheric and surface habitats, and recent advances have shed new insight on the mechanistic basis for this. Diverse microorganisms and biomolecules, including bacteria (particularly Gammaproteobacteria) (Joly *et al*. 2013), fungi (Fröhlich-Nowoisky *et al*. 2014), microalgae (Tesson and Šantl-Temkiv 2018) and microbially derived cell-free ice nucleating proteins (INpro) (O′Sullivan *et al*. 2015) can nucleate ice in super-cooled cloud droplets at temperatures much warmer than other atmospheric particles (between −13 and −1°C) and have been implicated in cloud processes. This property has been linked with specific surface- or excreted proteins and is essentially a biophysical process that occurs independently of cell viability or association (Fig. 6). Whilst terrestrial sources are dominant on a global level, marine sources associated with INpro excreted by marine microalgae (Wilson *et al*. 2015) may be important in remote regions unaffected by continents (e.g. Southern Ocean) (DeMott *et al*. 2016).

While ice nucleating bacteria are genetically diverse and have recently been proposed to employ different mechanisms for ice nucleation (Failor *et al*. 2017), among identified biological ice nucleation active molecules, the best understood are bacterial INpro. Recent advances have shed new insight on the mechanistic basis of their nucleation. These are large repetitive membrane-associated proteins (>120 kDa), which nucleate ice through a yet to be fully resolved mechanism (Pandey *et al*. 2016, Hudait *et al*. 2018, Lukas *et al*. 2020). They were recently found to structure interfacial water molecules at low temperature promoting ice formation (Roeters *et al*. 2021) (Fig. 6). Monomers or small oligomers nucleate ice at temperatures from −7 to −10°C and are known as Type C INpro; and higher order oligomers of INpro nucleate ice between −2 and −5°C and are known as Type A INpro (Wex *et al*. 2015, Ling *et al*. 2018). Laboratory studies using cloud simulation chambers have shown that the activity of bacterial INpro is independent of cell viability (Hartmann *et al*. 2013, Amato *et al*. 2015). Fungal spores and hyphae also carry INpro that exhibit different molecular structure and properties to those in bacteria and they are yet to be fully characterized. Fungal spores are the dominant state for fungi in the atmosphere and support surface-associated INpro and ice nucleating activity (Pouleur *et al*. 1992, Morris *et al*. 2013) (Fig. 6). Fungal hyphae occur very rarely in the atmosphere and so the major contribution arises from dissociation of fungal INpro from cell walls and adhesion to soil particulates that subsequently become aerosolized (O′Sullivan *et al*. 2015) (Fig. 6). The ubiquitous soil fungal genera *Fusarium* and *Mortierella* have been demonstrated to excrete INpro into the environment and soil particles with associated INpro were capable of ice nucleation (Fröhlich-Nowoisky *et al*. 2014, O′Sullivan *et al*. 2015).

Once they induce freezing, microbial INpro can also contribute to the formation of secondary ice particles in clouds that enhances the impact of low abundance biological INPs through ice multiplication, particularly at high sub-zero temperatures (Korolev and Leisner 2020) (Fig. 6). Rain that is produced via the ice phase through the Hallett–Mossop process strongly dominates over continents and is common over mid-latitude oceans (Mülmenstädt *et al*. 2015). This process occurs in mixed phase clouds, where some of the supercooled cloud droplets freeze due to the presence of ice nucleating particles. Water vapour is preferentially deposited on thus-formed ice particles and allows them to grow to precipitation sizes whilst being replenished by evaporating cloud droplets (Fig. 6). Ice nucleation active bacteria that are aerosolized from the phyllosphere were suggested to drive the bioprecipitation cycle by inducing rain formation. Rain enhances the growth of vegetation and epiphytic microorganisms, which in turn leads to enhanced emissions of ice nucleation active bacteria resulting in a positive feedback (Fig. 4D) (Morris *et al*. 2014). Bioprecipitation may be important above regions with a well-developed phyllosphere where emissions of bacterial INpro are highest, and accompanied by relatively low levels of abiotic INPs (Huffman *et al*. 2013, Healy *et al*. 2014). Other precipitation-related feedbacks that increase bioaerosol generation and thus enhance the atmospheric INpro include immediate and delayed interactions of rain with soil and plants (Huffman *et al*. 2013, Joung *et al*. 2017). These positive feedbacks occur alongside nonbiological ice nucleation and precipitation and the significance of these processes for meteorology and climate is yet to be quantified.

## How might anthropogenic activities affect atmospheric microbiota?

**Figure 7.**
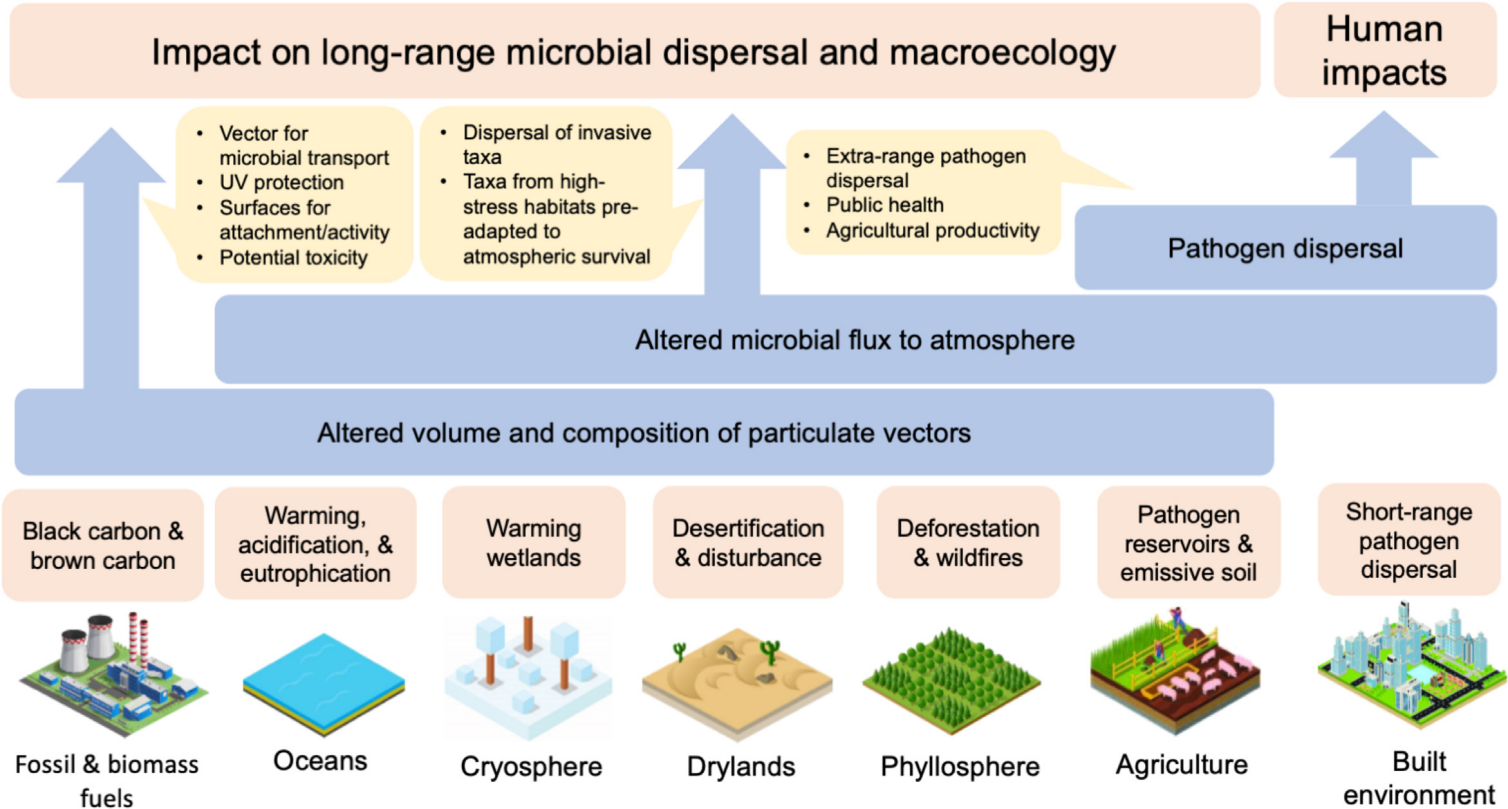
Potential anthropogenic impacts on atmospheric microbiology. Direct and indirect anthropogenic forcing of the atmosphere will potentially impact the emission and deposition flux, atmospheric burden, biodiversity, biophysical and metabolic activity of microorganisms in the atmosphere. We identify three main outcomes of anthropogenic forcing that are of potential concern, although these are likely inter-linked and overlap. They are altered atmospheric transport arising from changes to particulate matter (PM) burden in the atmosphere; shifts in atmospheric microbial emission and deposition flux due to change in surface microbial communities; and impacts on human systems due to agricultural and human pathogen dispersal. We advocate for future research focus to address these impacts.

The atmosphere is a primary sink for anthropogenic emissions of greenhouse gases, chemical pollutants and particulates from fossil fuel combustion (Archer and Pointing 2020). Additional particulate emissions occur as a result of biomass burning due to land clearance and wildfires, soil destabilization due to land use change and expansion of intensive agriculture, and climate change induced desertification (Archer and Pointing 2020). Most of these emissions are concentrated in the troposphere where long-range microbial transport, metabolism and biophysical transformations occur. Atmospheric forcing due to emissions has been acute in the industrial age and this has the potential to create positive and negative impacts on the dispersal outcomes for microorganisms transported in the atmosphere (Archer and Pointing 2020) (Fig. 7).

Gaseous emissions from fossil fuel combustion including SOx and NOx have been shown to inhibit cell function and survival of *Pseudomonas* strains isolated from clouds (Kondakova *et al*. 2016). Greenhouse gaseous, e.g. methane (Šantl-Temkiv *et al*. 2013b), can be metabolized by atmospheric bacteria. However, as *in situ* microbial activity and its influence on atmospheric chemistry is still not well constrained, it also remains unclear if elevated substrate levels in the atmosphere have the potential to significantly affect microbial activity and its outcomes (Archer and Pointing 2020). The ice nucleation activity of bioaerosols may also become more important to precipitation patterns in a warming world because they are more efficient at warmer temperatures from −10°C to 0°C, compared with mineral particulates (Christner *et al*. 2008a), and INpro emissions are likely enhanced by biomass burning and intensive agriculture (Moore *et al*. 2021). The potential impacts from direct and indirect anthropogenic particulate emissions are more apparent and widespread. Anthropogenic particulates including brown carbon from biomass combustion and black carbon from fossil fuel combustion account for ∼20% of global emissions (Chen *et al*. 2018). Mineral dust and soil emissions due to climate change impact on desertification (Pointing and Belnap 2014) and agricultural emissions (Salawu-Rotimi *et al*. 2021). Elevated concentrations of anthropogenic and desert dust particulates have been shown to influence atmospheric microbial diversity (Maki *et al*. 2019), and this may have implications for dispersal outcomes at deposition sites. The transport of microorganisms in plumes of airborne particulates is envisaged to provide additional protection from UV damage during atmospheric transport (González-Toril *et al*. 2020). Particulates arising from combustion are more hygroscopic than natural mineral particles and whilst this may be viewed as potentially facilitating greater survival and microbial activity, it must be balanced against the often toxic levels of PAH associated with these particulates (Hayakawa *et al*. 2018). Anthropogenic forcing through particulate emissions may also affect ice nucleation patterns in clouds and thus impact microbial flux and deposition in the atmosphere more generally through changes in precipitation.

A recent modelling study suggests that overall microbial diversity in the atmosphere may decline under future climate scenarios and this may severely impact ecosystem connectivity and function (Ontiveros *et al*. 2021). Conversely, the magnitude of emissions may increase due to the effects of climate-change forcing or pollution, e.g. due to marine phytoplankton blooms (Lewis *et al*. 2020) or desertification (Pointing and Belnap 2014). Strengthened emissions from point sources are anticipated for pathogenic microorganisms, antibiotic resistance genes and allergens, e.g. wastewater treatment plants (Han *et al*. 2020), livestock facilities (Zhao *et al*. 2014) and urban centres (Brodie *et al*. 2007, Woo *et al*. 2013). These emissions have direct public health relevance although dispersal appears to be localized. However, recent long-term studies of microbiota in rain and snow deposited at high altitude above the atmospheric boundary layer identified up to 3% of the total airborne microbiota as potential pathogens (Triadó-Margarit *et al*. 2022) and observed long-range transport of antibiotic resistance genes indicative of diffuse distribution related to agricultural sources (Caliz *et al*. 2022). Assessing the extent of anthropogenic impacts remains somewhat speculative as more dedicated studies are needed both to provide a better constrained understanding of current processes and to investigate impacts of highly complex anthropogenic changes to the atmospheric microbiota.

## Concluding remarks

The discipline of aeromicrobiology has benefitted enormously from recent advances in methodology that overcome the limitations associated with the study of ultra-low biomass habitats, and we view this is beneficial in a wider sense to improved ecological understanding of how the atmosphere links to surface terrestrial and marine biomes. Specifically, the ability to accurately estimate microbial diversity in air through rigorous decontamination of environmental sequence data has revealed that the atmospheric boundary layer supports a highly diverse but nonrandomly assembled microbiota. Atmospheric microbial assemblages are taxonomically structured reflecting strong environmental filtering, as well as distinct spatio-temporal biogeographic signals indicating a complex interplay of local and regional recruitment to the atmosphere. The long-held ecological view of atmospheric transport as neutral to dispersal outcomes is therefore no longer valid. Thus, the assumptions about how atmospheric microbial transport impacts community assembly and biogeography need to be revised. Knowledge gaps in this regard include high-quality quantitative data on fluxes between surface environments and the atmosphere, as well as improved understanding of postdepositional processes. Methodological improvements that directly address the problem of contamination in ultra-low biomass air samples from higher altitudes will also help resolve the vertical distribution of microorganisms in the atmosphere.

In the absence of evidence for widespread metabolic activity that influences biogeochemical outcomes, lack of microbial interactions and food webs or evidence for cell proliferation, we conclude that microbial biochemical transformations make a very limited contribution to overall atmospheric chemistry although they may be important in localized habitable areas such as clouds or plumes of hydrated particles. The notion of a metabolically active atmospheric microbiome must therefore be contemplated with a high degree of caution. The molecular mechanisms underpinning biogenic ice nucleation are becoming better resolved for bacterial INpro but are still lacking for other microbial groups. Given that the temperature range at which biogenic ice nucleation occurs is relevant in the context of a warming climate, we urge greater focus on microbial ice nucleation to predict its future impacts on the water cycle. Development of improved laboratory simulations for atmospheric conditions and *in situ* measurements will assist with further understanding of atmospheric microbial metabolic and biophysical activity and its boundaries in air.

In a broader sense, estimates of the pan-global microbiota and its ecological relevance require greater consideration of biomass in the gas-phase atmospheric microbial environment alongside solid-phase terrestrial and liquid-phase aquatic habitats. The increasing threats to environmental, organismal and human health due to changes in atmospheric transport of microorganisms because of anthropogenic forcing will be more clearly understood when empirical considerations of the atmospheric microbiota are incorporated into analytical frameworks, including the One Health and Planetary Health concepts. We therefore urge expansion of atmospheric microbiological monitoring and inclusion as part of global-scale atmospheric data networks.
